# EEG microstate architecture does not change during passive whole-body accelerations

**DOI:** 10.1007/s00415-020-09794-4

**Published:** 2020-05-28

**Authors:** M. Ertl, M. Schulte, M. Dieterich

**Affiliations:** 1 Department of Neurology, Ludwig-Maximilians-Universität München, Marchioninistraße 15, 81377 Munich, Germany; 2 Department of Psychology, University of Bern, Bern, Switzerland; 3 German Center for Vertigo and Balance Disorders (DSGZ), Ludwig-Maximilians-Universität München, Munich, Germany; 4 Munich Cluster for Systems Neurology (SyNergy), Munich, Germany

Dear Sirs,

EEG microstates are defined as brief periods during which the overall scalp topography remains stable. Only four distinct microstates, explaining up to 80% of the variance in rest EEG, were consistently found in microstate studies [1, 2]. Additionally, an association between the EEG microstates and resting state fMRI likely exists [3, 4]. The link between the four canonical microstates (A, B, C, D) and sensory manipulations has been addressed, but a coherent theory has not yet emerged. For example, the temporal parameters of microstate B could be altered by manipulation of the visual input [5].

The link between microstate B and the visual system [5] motivated us to investigate whether an association exists between one of the microstates and vestibular stimulation. This is of special interest, since a close interaction between the different sensory systems is known from fMRI studies [6], e.g., showing a change of the interaction mode with the shift of the dominance from one system to the other for the visual and somatosensory systems [7]. Compared to other sensory systems, the vestibular system possesses a few unique features. For example, natural stimulation of the system always causes a multimodal stimulation of multiple sensory systems [8]. Vestibular information is processed by a distributed cortical network represented in both hemispheres [9–11] with a preponderance in the non-dominant hemisphere [12]. Additionally, a reciprocal inhibitory interaction between the vestibular and the visual system was demonstrated [13].

Here, we investigated whether passive whole-body movements with weak to moderate acceleration intensities influence the overall microstate architecture in healthy participants sitting on a chair on a motion platform. The motivation for the experiment was to test whether passive body accelerations, which are mostly but not solely [8] sensed by vestibular input, have a similar impact on microstates as visual input.

The EEGs of 29 healthy volunteers (12 female, 17 male; 26.7 years ± 5.59 SD) were analyzed during passive body translations along the three main axes (fore/aft, left/right, up/down) generated by a motion platform (Moog©-6DOF2000E) and compared to the static rest condition. Sinusoidal profiles with a frequency of 0.5 Hz and an amplitude of 3 cm were used. The stimulation duration along every axis was 35 s. Subjects were instructed to keep their eyes closed and stay awake.

**Fig. 1:**
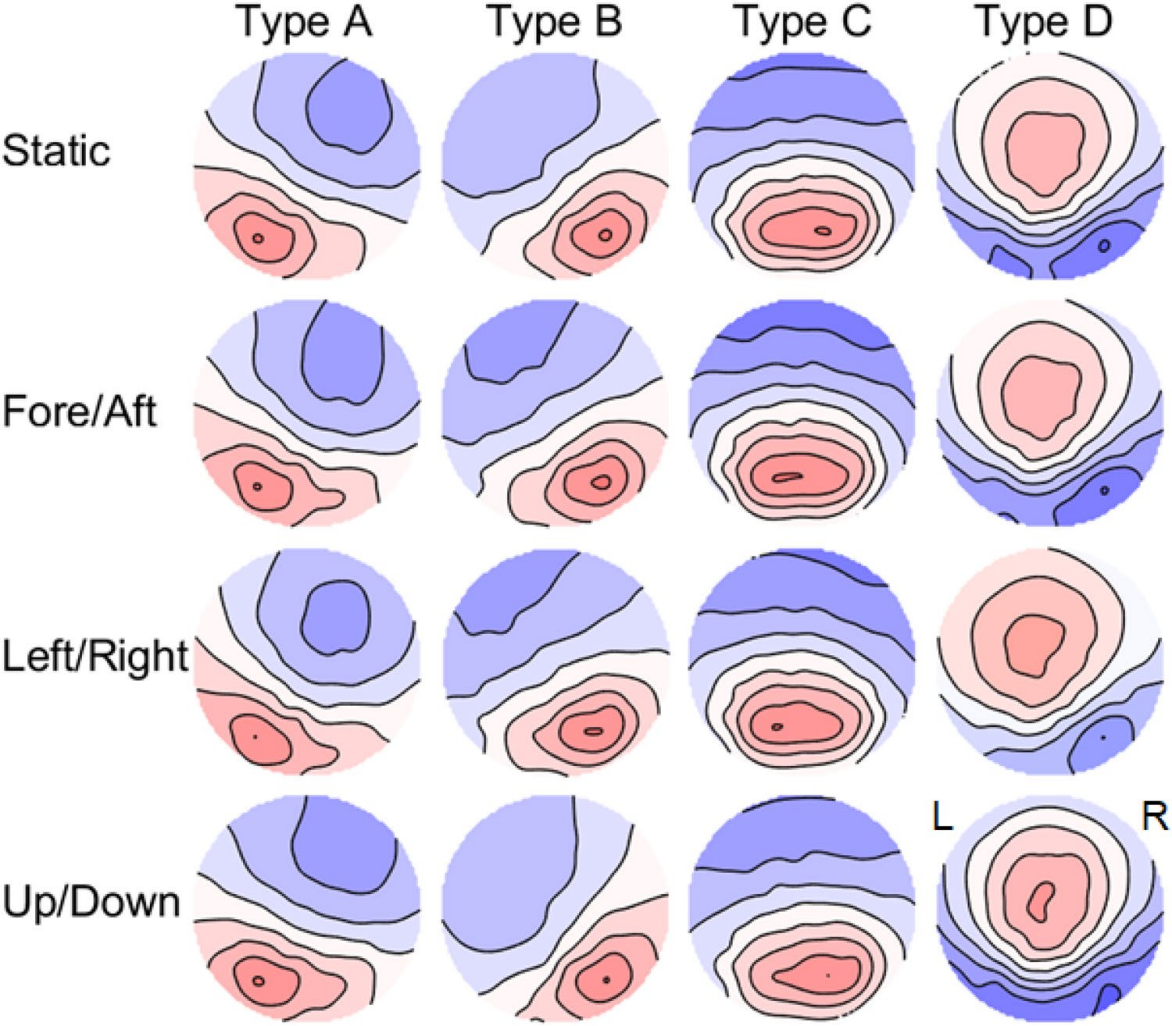
The topographies (head seen from above, nose up, left ear on the left) of the four microstate types (A–D) retrieved from the clustering algorithm for the four different motion conditions (static; fore/aft; left/right; up/down). The four types resemble the topographies reported by other studies and were sorted accordingly

The microstate analysis was performed in Matlab (Mathworks) using the EEGlab plug-in MicrostateAnalysis (Version 0.3, Thomas König). The data were band-pass filtered (2–20 Hz) and visually inspected for artifacts. Segments contaminated with artifacts were removed. The data were clustered within subjects using the widely used ‘atomize and agglomerate hierarchical clustering’ (AAHC) algorithm [14] and by ignoring polarity. Averages across subjects were calculated for any of the four movement conditions (Fig. 1), and a grand average across the conditions was computed. The statistical values for duration, occurrence, contribution, and explained variance were extracted and a between condition ANOVA was calculated for any of the four movement conditions.

The microstates obtained for the four conditions resembled the spatial distributions of the four canonical microstates and showed high inter-condition similarities (Fig. 1). The explained variance of the microstates was 76.3% across all conditions with no significant difference between the conditions (*F*(3,112) = 0.37, *p* = 0.777). This explained variance was well within the range (65–84%) typically reported in microstate analyses [1]. The average mean duration, defined as the average length of time a certain microstate remains stable whenever it appears, of the microstates were A: 67.1 ms, B: 70.2 ms, C: 67.7 ms, and D: 69.6 ms and therefore approximately 10 ms shorter compared to previous reports using an eyes-closed resting condition [15].

The mean contributions are the relative portions of the total time spent in any of the four microstates. In our data, the contributions of the four states were A: 25.5%, B: 24.7%, C: 24.9%, D: 24.9%. We also analyzed the average frequency of observation of the four microstates per second, which is called occurrence. The mean occurrences were A: 3.69/s, B: 3.46/s, C: 3.70/s, D: 3.58/s. No significant differences between the conditions were found for any of the three metrics. Thus, our analyses showed a smaller variance between the microstates with respect to duration, occurrence, and contribution compared to previous reports [15].

In conclusion, our results show that the EEG microstate architecture is, contrary to visual stimulation, invariant with respect to weak whole-body accelerations. To date, the relevance of the temporal structure as well as the correct number of microstates, their interpretation and the relationship between EEG microstates and the resting state networks measured by fMRI are only partially understood [1–3]. Future studies on microstates might reveal valuable insights, e.g., when comparing patients suffering from vestibular failure or functional dizziness and healthy controls, or when stronger vestibular stimuli are used.
